# Long-term results and PSA kinetics after robotic SBRT for prostate cancer: multicenter retrospective study in Korea (Korean radiation oncology group study 15–01)

**DOI:** 10.1186/s13014-018-1182-z

**Published:** 2018-11-23

**Authors:** Younghee Park, Hae Jin Park, Won Il Jang, Bae Kwon Jeong, Hun-Jung Kim, Ah Ram Chang

**Affiliations:** 1 Department of Radiation Oncology/CyberKnife Center, Soonchunhyang University Seoul Hospital, Daesagwan-ro 59, Youngsan-gu, Seoul, 04401 Republic of Korea; 2 Department of Radiation Oncology, Hanyang University College of Medicine, Seoul, Republic of Korea; 3 Department of Radiation Oncology, Korea Institute of Radiological and Medical Sciences, Seoul, Republic of Korea; 4 Department of Radiation Oncology, Gyeongsang National University School of Medicine and Gyeongsang National University Hospital, Jinju, Republic of Korea; 5 Department of Radiation Oncology, Inha University Hospital, Incheon, Republic of Korea

**Keywords:** Prostate cancer, Stereotactic body radiotherapy, PSA, Kinetics

## Abstract

**Background:**

To evaluate the treatment outcome and prostate-specific antigen (PSA) change after stereotactic body radiotherapy (SBRT) for localized prostate cancer.

**Methods:**

Patients with localized prostate cancer treated with SBRT at three academic hospitals were enrolled. Treatment was delivered using Cyberknife with dose range from 35 to 37.5 Gy in 5 fractions. Biochemical failure (BCF) was assessed with Phoenix definition and toxicities were scored with Radiation Therapy Oncology Group (RTOG) toxicity criteria. The PSA kinetics were analyzed in patients who received no androgen deprivation therapy (ADT) and showed no recurrence.

**Results:**

Of the total 88 patients, 14 patients (15.9%) received ADT. After median follow-up of 63.8 months, the 5-year BCF free survival (BCFFS) was 94.7%. Two patients experienced late grade ≥ 3 GI toxicities (2.2%). The median nadir PSA was 0.12 ng/mL (range, 0.00–2.62 ng/mL) and the median time to nadir was 44.8 months (range, 0.40–85.7 months). Patients who reached nadir before 24 months showed poorer BCFFS than the others. The rate of PSA decline was maximum in the first year after treatment and gradually decreased with time. The pattern of PSA change was significantly different according to the risk groups (*p* = 0.011) with the slope of − 0.139, − 0.161 and − 0.253 ng/mL/month in low-, intermediate- and high-risk groups, respectively.

**Conclusion:**

SBRT for localized prostate cancer showed favorable efficacy with minimal toxicities. The time to PSA nadir was significantly associated with treatment outcome. PSA revealed rapid initial decline and slower decrease with longer follow-up and the patterns of PSA changes were different according to the risk groups.

## Background

It has been clearly demonstrated that radiation dose-escalation increased the tumor control probabilities in prostate cancer [1, 2]. However, there are limits for radiation dose-escalation because increased radiation dose resulted in increased probability of normal tissue toxicities.

Studies have found that prostate cancer cells have a low α/β ratio of around 1.5 [3–5]. Generally, normal tissues such as bladder and rectum are known to have α/β ratio of 3 and prostate cancer cells are more sensitive to high radiation dose per fraction than normal tissues. Therefore, hypofractionated radiotherapy could deliver higher biological equivalent dose (BED) to cancer cells without increasing the BED to normal tissues. Hypofractionated radiotherapy with dose of 2.4–3.4 Gy per fraction has been evaluated in multiple studies. The efficacy of these moderate hypofractionation was not inferior to conventional fractionation with comparable toxicities [6–8].

Stereotactic body radiotherapy (SBRT), which delivers high dose of radiation in few fractions, is widely used in cancer treatment [9, 10]. SBRT very precisely delivers higher dose per fraction than in moderate hypofractionation and theoretically, SBRT could treat prostate cancer more effectively. Studies have reported the favorable outcome and acceptable toxicities of SBRT for localized prostate cancer [11–14].

Serum PSA is a well-established tumor marker for screening prostate cancer and monitoring response after treatment. The PSA change after radiotherapy has been extensively studied and several parameters such as PSA nadir, time to nadir or PSA velocity have been proposed as predictive factors for treatment outcome [15–18] . However, the PSA kinetics after SBRT have not been fully studied during long-term follow-up period.

In this study, we evaluated the long-term outcome of SBRT for prostate cancer and assessed the PSA kinetics after SBRT.

## Methods

### Patients

Patients with localized prostate cancer treated with SBRT from 2008 through 2014 at three academic hospitals in Korea were enrolled in this study. The inclusion criteria were as follows: 1) age ≥ 20 years; 2) histologically confirmed prostate cancer; 3) no regional lymph node metastases on abdominal CT and no distant metastasis on chest imaging (chest X-ray or CT) and bone scan; 4) SBRT with radical aim; 5) completion of planned SBRT; and 6) follow-up of ≥12 months. Patients with prior history of radiotherapy to pelvis or undergoing SBRT as boost treatment following whole pelvic radiotherapy were excluded. The medical records of included patients were retrospectively reviewed according to the study protocol approved by Korea Radiation Oncology Group (KROG) and the Institutional Review Board of each participant institutions.

### Treatment

SBRT was delivered by Cyberknife (Accuray, Inc., Sunnyvale, CA, USA). Gold fiducial makers were placed in the prostate for real-time motion tracking during treatment. The prescription dose was 35–37.5 Gy in five fractions on consecutive days (QD) or every other day (QOD). Treatment planning was performed using CT scan fused to MRI images. The clinical target volume (CTV) include prostate and seminal vesicles depending on the risks and margin of 3–5 mm was added to CTV to create the planning target volume. The prescribed dose was normalized to 75–85% isodose line. Less than 1 mL of rectum received 36 Gy and volume of bladder receiving at least 37.5 Gy was ≤5 mL.

### Follow-up and analysis

The patients were classified into prognostic risk groups according to the National Comprehensive Cancer Network (NCCN) guidelines (low-risk: clinical stage T1-T2a and Gleason score ≤ 6 and PSA < 10 ng/mL, intermediate-risk: clinical stage T2b – T2c or Gleason score 7 or PSA 10-20 ng/mL, high-risk: clinical stage T3a – T4 or, Gleason score 8–10 or PSA < 20 ng/mL). Biochemical failure (BCF) was defined using the Phoenix definition (nadir + 2 ng/ml). To assess the accurate effect of SBRT on PSA change, patients who received ADT were excluded from the analysis for PSA kinetics. Additionally, patients who experience recurrence were excluded when evaluating the PSA change in each risk groups to eliminate the compounding effect of PSA elevation in recurred patients. Acute and late toxicities were assessed with Radiation Therapy Oncology Group (RTOG) criteria.

All statistical analyses were performed using SPSS version 18.0 (SPSS Inc., Chicago, IL). BCF free survival (BCFFS) were calculated with Kaplan-Meier Methods. The log-lank test and Cox proportional-hazard model were used for univariate and multivariate analyses. The differences of PSA changes after SBRT between risk groups were evaluated with generalized estimating equations.

## Results

### Patient characteristics and treatment

A total of 88 patients were enrolled in this study. The baseline patient characteristics are shown in Table 1. The median age of patients was 69.5 years (range, 47–81 years). The median initial PSA was 6.95 (range, 2.05–23.04) ng/ml and most patients (98.9%) had disease confined to prostate (clinical stage ≤T2c). Fifty-six (63.7%) patients had Gleason score 6 or less. According to the NCCN guidelines, 24 (27.3%), 50 (56.5%) and 14 (15.9%) patients were in low-, intermediate- and high-risk group, respectively. Fourteen patients (15.9%) received neoadjuvant, concurrent or adjuvant ADT. Most of the patients (71.6%) received 37.5 Gy and the treatment was delivered on consecutive days in 54 (62.5%) patients (Table 2).

**Table 1:** Patient characteristics

	n	%
age, years		
median	69.5	
range	47–81	
initial PSA, ng/ml		
median	6.95	
range	2.05–23.04	
preCK PSA, ng/ml		
median	6.65	
range	0.01–21.62	
T stage		
1c	27	30.7
2a	19	21.6
2b	14	15.9
2c	27	30.7
3a	1	1.1
Gleason score		
≤ 6	56	63.7
7	20	22.7
≥ 8	12	13.6
Risk group		
low	24	27.3
intermediate	50	56.5
high	14	15.9
ADT		
no	74	84.1
yes	14	15.9
Prostate volume, ml		
median	40.0	
range	12.0–94.8	
Initial IPSS		
Median	3	
range	0–15	

*Abbreviations*: *PSA* Prostate-specific antigen, *ADT* Androgen deprivation therapy, *IPSS* International prostate symptom score

**Table 2:** Summary of SBRT dose and treatment schedule

	n	%
Total dose (Gy)		
35.00	2	2.3
36.25	22	25.0
36.50	1	1.1
37.50	63	71.6
Treatment schedule		
QD	55	62.5
QOD	33	37.5

*Abbreviations*: *QD* Daily treatment, *QOD* Every-other-day treatment

### Treatment outcome and PSA kinetics

#### Treatment outcome in all patients

**Fig. 1:**
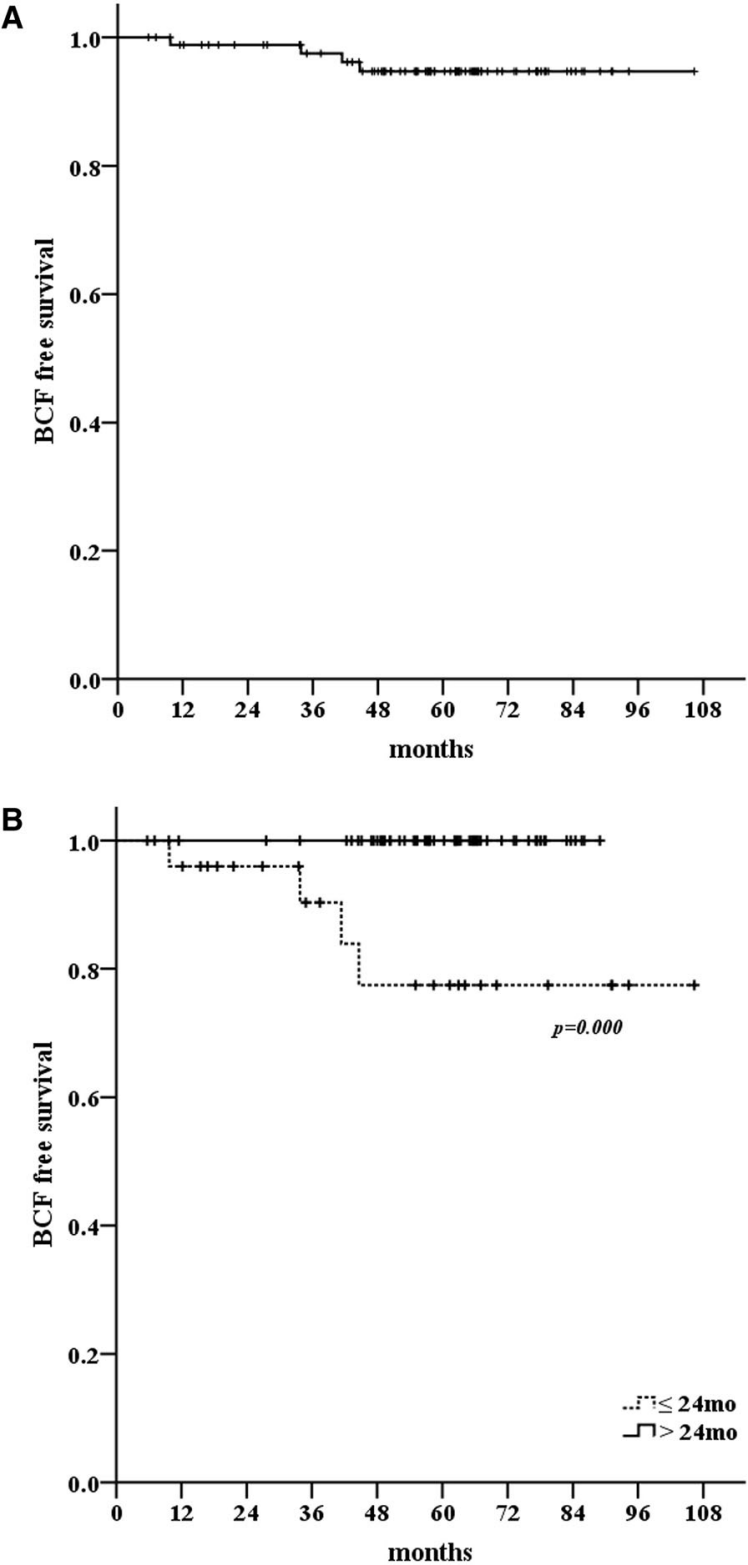
The biochemical failure-free survival in all patients (**a**) and according to the time to nadir (**b**)

After median follow-up of 63.8 months (range 12.1–109.5 months), 4 patients experienced a biochemical relapse (2 low-, 1 intermediate- and 1 high-risk group). One of the recurred patients received concurrent ADT. The actuarial 5-year BCFFS was 94.7% (Fig. 1-a). The median nadir PSA was 0.12 ng/mL (range, 0.00–2.62 ng/mL) and median time to nadir was 44.8 months (0.40–85.7 months) in all patients.

#### PSA kinetics in no ADT group

To exclude the effect of ADT on the PSA change, we excluded 14 patients who received ADT in the analysis for PSA kinetics. In 74 patients who did not received ADT, the median nadir PSA was 0.15 ng/mL (0.01–2.62 ng/mL) and median time to nadir was 47.3 months (1.2–85.7 months). The nadir PSA value did not show any significant association with treatment outcome in these patients. However, patients who reached nadir PSA 24 months after SBRT showed better BCFFS rate than the other patients (*p* = 0.001, Fig. 1-b).

**Fig. 2:**
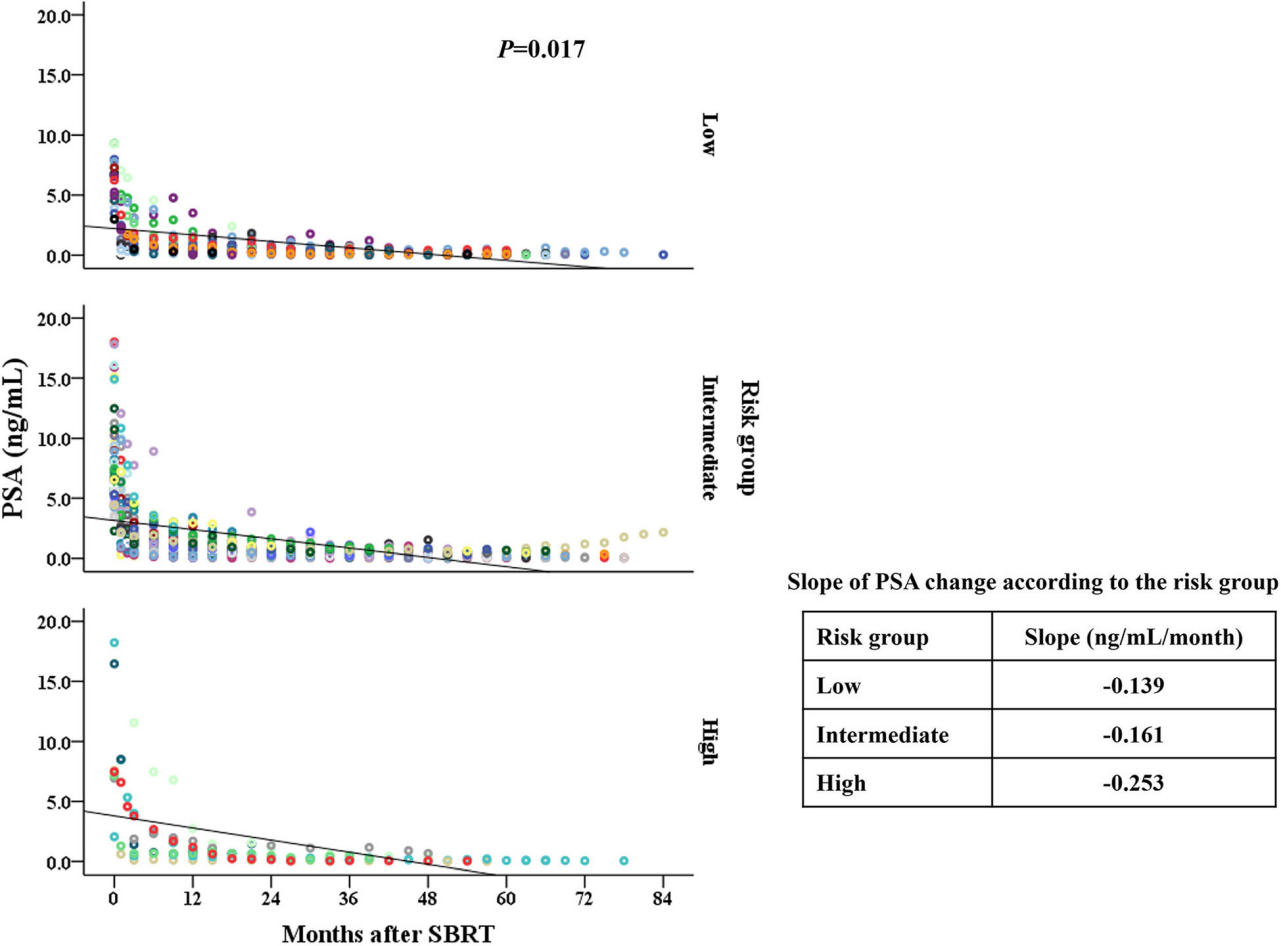
The PSA change after SBRT according to the risk groups. Circles of the same color represents the PSA values of one patient measured at each time point

To evaluate the PSA declining kinetics after SBRT excluding the effect of recurrence, additional 3 patients with recurrence were excluded. In the remaining 71 patients, the median nadir PSA value was 0.075, 0.23 and 0.13 ng/mL and median time to nadir was 53.4, 46.4 and 50.1 months after SBRT in low-, intermediate- and high-risk groups, respectively. There were no statistically significant differences in nadir value or time to nadir according to the risk groups. The benign PSA bounce was observed in 25 patients (35.2%) and the frequency of PSA bounce was not associated with risk groups or Gleason score. The median time to bounce was 11.0 months (range, 2.9–38.5 months) after SBRT, and the median height of PSA bounce was 0.53 ng/ml (range, 0.24–2.62 ng/ml). The rate of PSA decline was maximum in the first year after treatment and decreased with time. The median slope of PSA decline was − 0.47, − 0.27, and − 0.18 ng/mL/month in the 1, 2, and 3 year after SBRT, respectively (Fig. 2). The slope of PSA change was significantly different according to the risk groups (*p* = 0.017) with the median values of − 0.139, − 0.161 and − 0.253 ng/mL/month in low-, intermediate- and high-risk groups, respectively. The total Gleason score or primary Gleason score did not affect the rate of PSA change after SBRT.

### Toxicity

There were no grade ≥ 3 acute gastrointestinal (GI) or genitourinary (GU) toxicity. (Table 3) No patients experienced grade ≥ 3 late GU toxicity but grade ≥ 3 late GI toxicity occurred in 2 patients (2.2%). One patient had grade 3 rectal bleeding and one developed grade 4 recto-urethral fistula, which required colostomy and cystostostomy at 19 months after SBRT. The treatment schedule (every-other-day treatment vs. daily treatment) did not affect the rate of acute or late toxicities.

**Table 3:** Acute and late toxicity

		GI			GU	
	Grade	n	%	Grade	n	%
Acute Toxicity	1	32	36.4	1	43	48.9
	2	5	5.7	2	8	9.1
Late Toxicity	1	10	11.4	1	27	30.7
	2	1	1.1	2	2	2.3
	≥3	2^a^	2.3			

*Abbreviations*: *GI* Gastrointestinal, *GU* Genitourinary

^a^Rectal bleeding and recto-urethral fistula

## Discussion

In this study, we found excellent treatment outcome with low rate of treatment related toxicity after SBRT in localized prostate cancer. The favorable outcomes of SBRT and acceptable toxicity rates have been reported in previous studies with follow-up of 2–3 years [11–13]. In our results, we only included patients with follow-up of more than 1 year and the median follow-up duration was 64.5 months providing favorable long-term results. Previously, two studies have reported long-term outcomes comparable to our study [19, 20]. Freeman et al. reported 5-year outcome of SBRT with 35–36.25 Gy / 5 fractions in 41 low-risk prostate cancers patients. The biochemical progression-free survival rate was 93%, which they suggest comparable to brachytherapy or surgery with less toxicity profiles. Katz et al. also reported the outcomes of a large study consisting of 304 patients with median follow-up of 60 months [20]. They performed the SBRT at 2 dose levels, 35 Gy or 36.25 Gy in 5 fractions, and found no effect of total dose on the treatment outcome or PSA nadir levels. Late urinary or rectal complication was more frequent in patients treated to 36.25 Gy but the difference was not statistically significant. They included all risk groups and the 5-year biochemical recurrence-free survival was 97, 90.7 and 74.1% for low-, intermediate- and high-risk patients, respectively.

Although SBRT has been accepted as a proper treatment option for low- to intermediate-risk prostate cancer patients, the efficacy in high-risk patients is unknown. We included 14 (15.9%) high-risk patients and could not find any significant difference in the outcome among different risk groups. The treatment outcome of high-risk patients in our study (3- and 5-year BCCFS, 100 and 91.7%) which were remarkably better than in study by Katz et al. In another study [21], Katz et al. reported the long-term outcome of SBRT for high-risk patients comparing the outcome of SBRT alone with pelvic radiation followed by SBRT boost and found no difference in outcome between two treatment groups. They used same radiation doses with previous study (35 and 36.25 Gy), and their outcome was still relatively poor (6-year biochemical disease-free survival, 69%) compared to results of other risk group. Again in that study, they found no dose response in both treatment groups. Oliai et al. reported the outcome of SBRT for all risk group of prostate cancer patients [13]. A total of 70 patients received SBRT at 3 levels of 35, 36.25 and 37.5 Gy in 5 fraction and outcomes of high dose (37.5 Gy) and low dose (36.25 and 35 Gy) groups were compared. As a result, they found a dose response in intermediate- and high-risk patients (3-year freedom from biochemical failure, 100% in high dose vs. 72% in low dose group, *p* = 0.0363). We also used the same high dose of 37.5 Gy as in a study of Oliai et al. in most high-risk patients (12 of 14 patients, 85.7%). The treatment outcome of high-risk patients in our study (3- and 5-year BCFFS, 100 and 91.7%) was also comparable to the high dose group of Oliai study. Additionally, the poor outcome of high-risk patient in a study of Katz et al. was similar to the outcome of low dose group of Oliai study. These results suggest that although SBRT showed favorable results in prostate cancer, higher dose of SBRT is needed and the dose as high as 37.5 Gy used in our study would be effective for high-risk groups. There has been dose escalation SBRT studies for low- to intermediate-risk groups and dose escalation to 50 Gy has been completed without dose limiting toxicity [22, 23]. A large and well-designed prospective study would be needed to find the optimal dose for high-risk prostate cancer.

The nadir PSA has been suggested as a significant prognostic marker in conventional fractionation radiotherapy by numerous studies and they proposed 0.2–1.5 ng/mL as a cut-off value for predicting the outcome [15, 24, 25]. Ray et al. reported PSA nadir and time to nadir were significant predictor for disease-free survival and distant metastasis-free survival [18]. They categorized the nadir PSA into 4 levels, < 0.50, 0.50–0.99, 1.0–1.99 and ≥ 2.00 ng/mL and found better survival with lower nadir PSA level. However, in our study, the nadir PSA did not show any association with BCFFS. The median value of nadir PSA in our study was 0.12 ng/mL and more than half of the patients (*n* = 54, 61.4%) reached nadir less than 0.2 ng/mL which is significantly lower than the reported nadir value of conventional radiotherapy. Previously, Kishan et al. reported that nadir PSAs after SBRT or HDR brachytherapy were significantly lower than IMRT [26]. Anwar et al. also reported the lower PSA nadir after SBRT than conventional fractionation radiotherapy [27]. Therefore, conventional threshold value for nadir to predict the outcome may not be useful in SBRT, which achieves much less nadir PSA.

After SBRT, the PSA showed maximum decline in the first year after SBRT and gradual decline continued until the last follow-up. At the same time, the time to nadir PSA also increased with longer follow-up. Similar PSA kinetics after SBRT have been reported in several studies [26–28]. They all reported the initial rapid decline of PSA followed by a prolonged slow decay. They suggested that rapid decline of PSA in initial phase is caused by destruction of malignant cells and further prolonged decrease reflects the decline of PSA produced by benign tissues. In our study, the time to nadir PSA showed significant association with BCFFS. Patient who achieved nadir PSA after 24 months showed better prognosis than those before 24 months. Fourteen patients who received neoadjuvant, concurrent or adjuvant ADT were excluded in this analysis to exclude the effect of ADT on the outcomes. In conventional fraction radiotherapy, Ray et al. reported the similar results [18]. They found that longer time to nadir PSA significantly associated with improved biochemical and distant failure-free survival. Generally, it is well known that prostate cancer shows heterogeneity in malignant potential and multiple Gleason grade can be found in the same specimen [29, 30]. Therefore, prostate cancer cells with diverse malignant potential might have diverse α/β ratio. As shown in our study with two-phase decline of PSA, the prostate cancer cells are assumed to have different destruction pattern after SBRT. Cancer cells with more malignant potential could be removed early after SBRT and less aggressive cancer cells with normal prostate cells could show more protracted death. Therefore, longer time to nadir PSA could reflect the presence of more indolent cancer cells and this could have resulted in better prognosis. The various reported α/β ratio values of prostate cancer could have resulted from this heterogeneity of prostate cancer [3–5].

We tried to evaluate PSA changes in different risk groups after SBRT. To eliminate the effect of recurrence on PSA decline, total number of non-recurred 71 patients were included in this analysis. As a result, the PSA change was significantly different according to the risk groups. High-risk group showed steepest slope of PSA decline. Total Gleason score, or primary grade alone, did not show any significant differences in PSA slope. Although we failed to find any relationship between risk groups and prognosis after long-term follow-up, we suggest that different risk groups have different ratio of more malignant and more indolent cancer cells and show different PSA decay pattern, which were well incorporated in the prognosis of prostate cancer. Further study in a prospective and well-stratified way regarding the heterogeneity of malignant potential in all the prostate cancer risk groups and PSA kinetics after SBRT can validate our hypothesis.

Our study has an inherent limitation that it is retrospective study. However, we included large numbers of patients from 3 academic institutions and provided clinical outcomes and changes of PSA values after longer follow-up (63.8 months).

## Conclusions

In conclusion, SBRT for prostate cancer is effective and safe even in all risk group patients. The longer time to nadir PSA was predictive for better outcome. PSA showed rapid initial decrease and continued gradual decline with longer follow-up and velocity of PSA decline was different according to the risk groups. These PSA kinetics including nadir value and time to nadir, which is distinctive from conventional radiotherapy, should be considered during the follow-up periods after SBRT for prostate cancer.

## Abbreviations

ADT: Androgen deprivation therapy
BCF: Biochemical failure
BCFFS: BCF free survival
BED: Biological equivalent dose
CTV: Clinical target volume
KROG: Korea Radiation Oncology Group
NCCN: National Comprehensive Cancer Network
PSA: Prostate-specific antigen
QD: Once a day
QOD: Every other day
RTOG: Radiation Therapy Oncology Group
SBRT: Stereotactic body radiotherapy
